# Mediation effect of stroke recurrence in the association between post-stroke lactate dehydrogenase and functional disability

**DOI:** 10.3389/fnagi.2024.1450863

**Published:** 2024-08-30

**Authors:** Qian He, Miaoran Wang, Haoyue Zhu, Ying Xiao, Rui Wen, Xiaoqing Liu, Yangdi Shi, Linzhi Zhang, Yu Wang, Bing Xu

**Affiliations:** ^1^Shenyang Tenth People’s Hospital (Shenyang Chest Hospital), Shenyang, China; ^2^Qionglai Traditional Chinese Medicine Hospital, Chengdu, China; ^3^China Medical University, Shenyang, China; ^4^Shenyang First People’s Hospital, Shenyang, China

**Keywords:** acute ischemic stroke, lactate dehydrogenase, stroke recurrence, modified Rankin scale, mediation analysis

## Abstract

**Background:**

We aimed to use lactate dehydrogenase (LDH) as a marker of inflammation burden and quantify post-stroke inflammation’s direct and indirect effect on functional disability.

**Methods:**

We analyzed 5,129 patients with acute ischemic stroke (AIS) admitted to Shenyang First People’s Hospital. Stroke recurrence and functional outcome measured by the modified Rankin Scale (mRS) were assessed at 90 days. Functional disability was defined as mRS score > 2. Receiver operating characteristic curve and restricted cubic spline (RCS) analysis were conducted to illustrate the associations between LDH levels and 90-day functional outcomes in patients with AIS. Mediation analyses were performed to examine the potential causal chain in which stroke recurrence may mediate the relationship between LDH and functional outcome. Positive correlation between LDH and hs-CRP was found and mediation effects of stroke recurrence in the association between LDH or hs-CRP and functional disability were both less than 20%. Sensitivity analyses in different subgroups showed comparable results.

**Results:**

Among 5,129 included AIS patients, the median (IQR) level of LDH was 186 (161–204.4) U/L. Functional disability was seen in 1200 (23.4%) patients and recurrence was observed in 371(7.2%) patients at 90-day follow-up. Each standard deviation increase in the concentration of LDH was linked to an increased risk of functional disability (adjusted odds ratio[aOR], 1.07; 95%CI,1.04–1.09) and stroke recurrence (aOR,1.02; 95%CI, 1.01–1.04) within 90 days. The highest quartile of LDH (>204.2 U/L) had an elevated risk of suffering functional disability (aOR, 1.21; 95%CI, 1.00–1.47) and recurrence (aOR, 1.21; 95%CI,1.00–1.47) compared with the lowest quartile of LDH (<161 U/L). Stroke recurrence during follow-up explained 12.90% (95%CI, 6.22–21.16%) of the relationship between LDH and functional disability. Positive correlation between LDH and hs-CRP was found and mediation effects of recurrence in the association between LDH or hs-CRP and functional disability were both less than 20%. Sensitivity analyses in different subgroups showed comparable results.

**Conclusion:**

The relationship between LDH and functional disability at 90 days among AIS patients is partially mediated by stroke recurrence, accounting for less than 20%. LDH deserves equal attention as hs-CRP in predicting recurrence and functional outcome. In addition to traditional secondary prevention measures, innovative anti-inflammatory strategies warrant further investigation.

## Introduction

Inflammation is widely recognized as playing a crucial role in the etiology and prognosis of ischemic stroke (Pei et al., 2015; Jin et al., 2013; Gu et al., 2022; Shekhar et al., 2018). Lactate dehydrogenase (LDH) has been identified as a non-specific biomarker of inflammation, with a positive correlation observed between LDH levels and both stroke recurrence and unfavorable outcomes in patients with acute ischemic stroke (AIS) (Jin X. X. et al., 2022; Wang et al., 2021; Jin H. et al., 2022). Additionally, evidences also showed that stroke recurrence was highly linked to poor prognosis (Xu et al., 2022; Wang A. et al., 2016; Ding et al., 2022).

LDH remains predominantly intracellular unless there is local tissue injury (Jin H. et al., 2022), leading to elevated serum levels following an AIS due to brain cell damage or death (Tian et al., 2020). The presence of LDH as a post-stroke inflammation biomarker is associated with cellular death, brain injury, and blood–brain barrier disruption (Jiang et al., 2018; Yang et al., 2019), all of which contribute to functional impairment (Doyle et al., 2008). Besides, LDH contributes to platelet aggregation (Milias et al., 2005) and intravascular thrombosis (Fattizzo et al., 2022), potentially leading to recurrent strokes and subsequent indirect functional deficits.

Currently, studies on the role of high sensitive C-reactive protein (hs-CRP) as a prognostic marker in AIS patients are frequently conducted (Cheng et al., 2023; Pu et al., 2022; Li et al., 2016; Lee et al., 2020), while those focused on serum LDH either independently or in conjunction with increased hs-CRP levels, has been comparatively neglected in the scientific discourse. It is still unknown, if LDH serves a similar function as hs-CRP in predicting functional outcome and recurrence and how much stroke recurrence mediates the association between elevated LDH levels and functional disability. Our hypothesis posits that follow-up stroke recurrence plays a mediating role in the association between LDH levels and functional outcomes at 90 days post-stroke. Among AIS patients, our study examined whether stroke recurrence mediated the relationship between LDH or hs-CRP levels and functional outcomes at 90 days, with exploring the extent of this mediation effect. We also evaluated the mediation effect in various key subgroups.

## Methods

### Study design and subjects

We retrospectively collected data from a single center. It is a stroke registry database, which contains consecutive patients with AIS and is attached to Shen Yang First People’s Hospital in Shen Yang, China. Patients with modified Rankin scale (mRS) ≤ 2 from January 1st, 2017 to September 30th,2023 were enrolled. Informed consent has been obtained. Patients with prior severe intracranial hemorrhage or large hemispheric infarction, patients with missing data on LDH or 90-day mRS scores and patients lost to be followed and patients who had undergone intravenous thrombolysis or thrombectomy were excluded from this analysis.

### Data collection

Baseline data contained demographics (age, gender); National Institutes of Health Stroke Scale (NIHSS) scores at admission; Activity of daily living (ADL) scores at admission; blood pressure; smoking status; drinking status; toast type and medical history including heart failure, previous heart disease, prior stroke; diabetes mellitus, hypertension, atrial fibrillation and hyperlipidemia. Blood samples were collected within 24 h of admission. The concentration of LDH was tested and determined by using Beckmann AU5800 automatic biochemical analyzer after standard serum extraction.

Missing values in baseline characteristics (<20%) were imputed, and a comparison between the original and imputed data was conducted, with the results annexed in Supplementary Table S1.

### Patient follow-up and outcomes

Patients were monitored for 90 days post-discharge and underwent interviews conducted by trained research coordinators using a standardized process either face-to-face or over the phone. Stroke recurrence was defined as the development of a new neurological deficit lasting more than 24 h or subsequent hospitalization with a diagnosis of intracerebral hemorrhage, subarachnoid hemorrhage, or ischemic stroke. MRS scores (range of 0–6, with 0 indicates no disability and higher values indicate more severe disability) was used to measure functional outcomes; mRS >2 was seen as functional disability.

### Statistical analysis

Baseline characteristics were compared and described by quantiles of LDH. Continuous variables adhering to a normal distribution were characterized by their means alongside standard deviations (SD), and were subjected to analysis of variance (ANOVA) to assess statistical differences. For those continuous variables that deviated from normality, the median and interquartile ranges (IQRs) were reported, and comparisons were made employing the Kruskal-Wallis test. Categorical variables were presented as frequencies and percentages and were tested using chi-square tests. The detailed distribution of LDH concentration was presented using Kernel density estimation. Receiver operating characteristic (ROC) curve of LDH predicting 90-day functional outcome was created, with presenting the value of area under curve. The concentration of LDH was first taken as a categorical variable with Q1 as the reference group; then, it was treated as a continuous variable with increments of I standard deviation in logistic regression models to measure the associations between LDH and stroke recurrence or functional outcome at 90 days by odds ratios (ORs) and 95% confidence intervals (CIs). In addition, a four-node restricted cubic spline was used to test the nonlinear relationship between the percentiles of the LDH distribution (25th, 50th, and 75th) and the dose–response relationship of the result.

**Figure 1:**
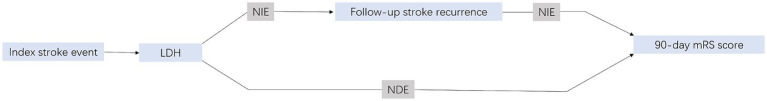
Illustration of mediation effect. mRS, indicates modified Rankin scale; NDE, natural direct effect; NIE, natural indirect effect. Total effect = NDE + NIE.

In order to clarify the relationship between LDH and functional outcome, we assessed both direct associations unaffected by stroke recurrence and indirect associations influenced by stroke recurrence as a mediating factor (Figure 1). Utilizing a causal mediation analysis within a counterfactual framework which offers a broad framework with precise definitions of casual mediation and related effects (Richiardi et al., 2013; VanderWeele, 2016; Valeri and Vanderweele, 2013), we were able to delineate the total effect (TE) into the natural direct effect (NDE) and the natural indirect effect (NIE), quantifying each as an odds ratio (OR). This approach provided a comprehensive understanding of causal mediation and related effects. The NIE captured the impact of LDH on functional disability that could be attributed to changes in the status of follow-up stroke recurrence, while the NDE represented the impact of LDH on functional disability that was not influenced by stroke recurrence. The Percentage Mediated (PM), which quantifies the proportion of the total effect that is mediated by the mediator, was calculated as (NIE/TE) * 100% on a log-transformed odds ratio scale (Vanderweele and Vansteelandt, 2010). Within this framework, two multivariate logistic regression models were meticulously crafted: the first to assess functional disability (outcome), predicated on levels of LDH, stroke recurrence, and a comprehensive array of study confounders, and the other for stroke recurrence (mediator) conditional on LDH (exposure) and all study confounders. Confounders in this analyses were factors known to be related to the recurrent stroke and functional prognosis, including demographics (age, gender), mRS score at admission, NIHSS scores at admission, ADL scores at admission, systolic blood pressure, diastolic blood pressure, smoking, drinking and medical history (heart failure, previous heart disease, diabetes mellitus, hypertension, atrial fibrillation, hyperlipidemia, prior stroke) and TOAST subtypes.

**Figure 2:**
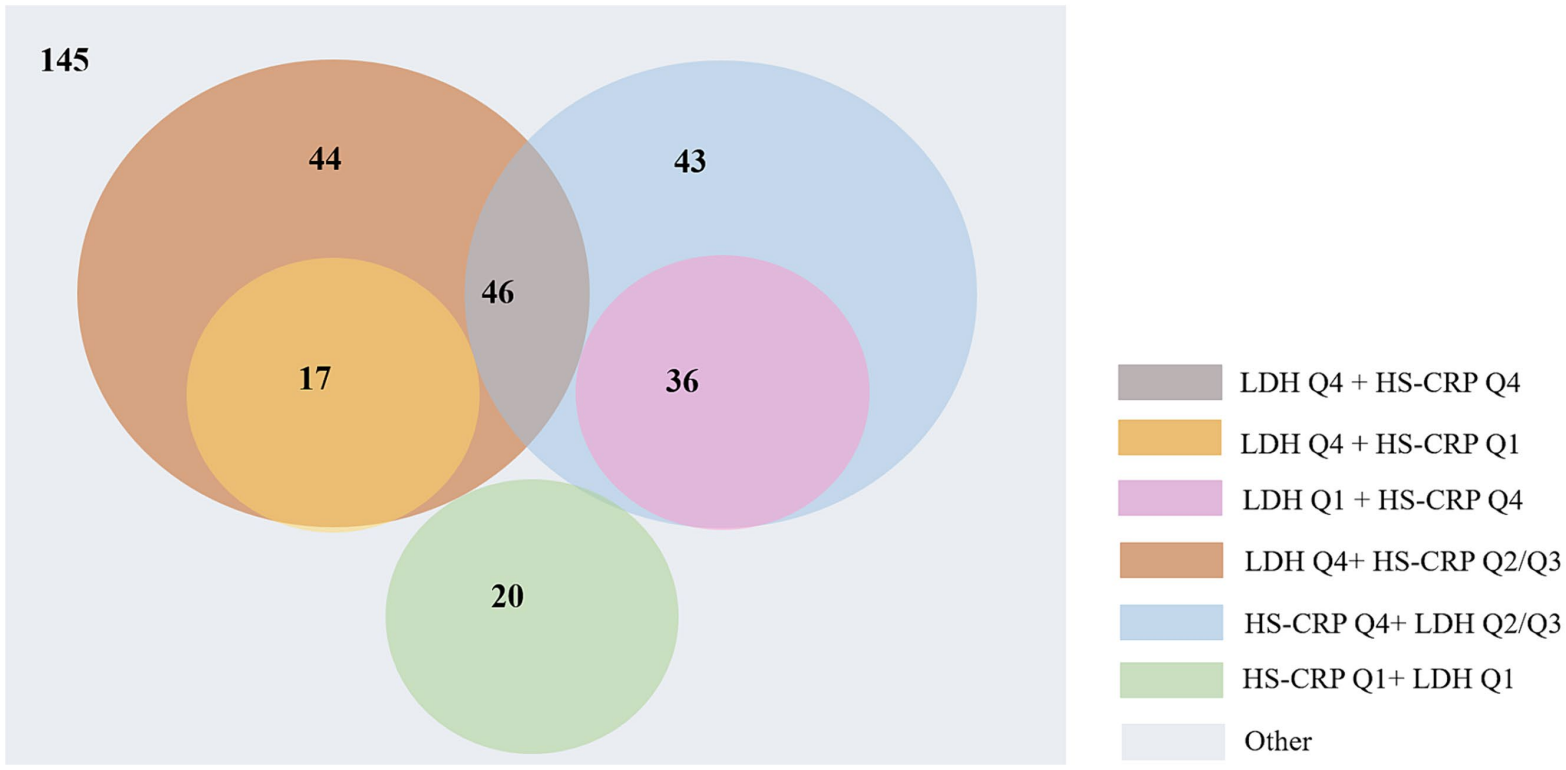
The detailed distribution of 351 AIS patients with functional disabilities based on LDH and hs-CRP quartiles.

To elucidate the mediating effect more clearly and specifically, we conducted a more in-depth analysis. Initially, we performed a correlation analysis between LDH and high sensitive C-reactive protein (hs-CRP) from 1,258 out of 5,129 AIS patients who were tested for both LDH and hs-CRP, with calculating Pearson’s correlation coefficient. Subsequently, LDH and hs-CRP were categorized into quartiles based on their concentrations (25th, 50th, and 75th), and the functional disability rates within all patients with functional disability (351 individuals) were calculated, specifically for those in the highest quartile (Q4) of LDH, those in the highest quartile of hs-CRP (Q4), and the union of both Q4 groups. Chi-square tests were used to compare the differences. Furthermore, from the 351 patients with functional disability, we selected 119 individuals who were in either the lowest quartile (Q1) or the highest quartile (Q4) for both LDH and hs-CRP. The functional disability rates were calculated for the following groups: LDH Q1 and hs-CRP Q1, LDH Q1 and hs-CRP Q4, LDH Q4 and hs-CRP Q1, and LDH Q4 and hs-CRP Q4. Chi-square tests were used to compare the differences (Figure 2). Then, LDH and hs-CRP were treated as continuous variables, and using the same cohort of 1,258 AIS patients, we conducted two mediation analyses with LDH and hs-CRP as independent variables and functional disability as the dependent variable, and stroke recurrence as the mediating variable. All analysis were performed with R statistical software (version 4.2.2; R Foundation for Statistical Computing, Vienna, Austria). *p* < 0.05 (2-tailed) were deemed significant.

### Sensitivity analyses

To test the robustness of our analysis, a series of sensitivity analyses were conducted. First, the mediation effect was recalculated after excluding cases that had died prior to stroke recurrence to mitigate the influence of competing risks of death. Then, we conducted causal mediation analysis stratified by age (≤65,>65), smoking (yes, no), gender (female, male), smoking status (yes, no), heart failure (yes or no), drinking (yes, no), previous heart disease (yes, no), hypertension (yes, no), atrial fibrillation (yes, no), ischemic heart disease (yes, no), hyperlipidemia (yes, no), prior stroke (yes, no), TOAST type (LAA, CE, SAA, SOE), NIHSS scores at admission (<3, ≥3) and ADL scores at admission (≤60, >60), since the functional outcomes may be impacted by the severity of the ischemic stroke and the subtype of ischemic stroke.

### Ethics statement

The research was granted approval by the Ethics Review Committee of Shenyang First People’s Hospital [2023SYKYPZ56], and all methods were performed in accordance with the relevant guidelines and regulations. Patient data was sourced from the hospital’s database, with each patient being duly informed about the utilization of the database and having provided their consent (In our hospital, for clinical data, laboratory data and treatment results are used for research purposes, admitted patients are invited to give pan-informed consent and sign written informed consent). The corresponding author processes access to all pertinent information.

## Results

A total of 8,038 AIS patients with mRS ≤ 2 at admission who did not receive thrombolysis or thrombectomy therapy were included in our study. After the further exclusion of 2,909 patients without 90-days mRS, had missing data on LDH, or lost to follow-up, 5,129 patients were included in this analysis (Supplementary Figure S1). Patients included and excluded from this analysis were largely comparable (Supplementary Table S2).

Among 5,129 patients, the mean [SD] age was 64.97[10.51] years old; 3,632 (71%) were men; 2,234 (43%) patients smoked and 1,503 (29%) drank; the median NIHSS score at admission were 3.0 (2.0—5.0) and the median ADL scores at admission was 80 (60–95); the mean [SD] systolic blood pressure and diastolic blood pressure were 149.61[19.73] and 87.04 [12.66], respectively. The most common previous medical condition was hypertension (67% [*n* = 3,458]), followed by prior stroke (36% [*n* = 1824]) and diabetes mellitus (32%, [*n* = 1,616]). Only 98 (2%) patients had heart failure and 340 (7%) had atrial fibrillation. More than three-quarter of strokers with LAA (Table 1).

**Table 1:** Baseline characteristics of AIS patients in by quartile of LDH at admission.

Variables	Total (*n* = 5,129)	Quartile 1 (*n* = 1,312)	Quartile2 (*n* = 1,289)	Quartile 3 (*n* = 1,235)	Quartile 4 (*n* = 1,293)	*p* value
LDH level, U/L	186 (161,204.2)	<161	161–186	186–204.2	>204.2	
**Demographic**						
Age	64.97 ± 10.51	63.44 ± 9.81	65.23 ± 10.35	65.05 ± 10.36	66.18 ± 11.28	< 0.001
Gender, *n* (%)						< 0.001
Male	3,632 (71)	1,036 (79)	944 (73)	860 (70)	792 (61)	
Female	1,497 (29)	276 (21)	345 (27)	375 (30)	501 (39)	
Smoking, *n* (%)	2,234 (44)	674 (51)	589 (46)	519 (42)	452 (35)	< 0.001
Drinking, *n* (%)	1,503 (29)	446 (34)	370 (29)	383 (31)	304 (24)	< 0.001
NIHSS scores at admission	3.0 (2.0–5.0)	3.0 (2.0–5.0)	3.0 (1.0–5.0)	3.0 (1.0–5.0)	3.0 (2.0–6.0)	0.002
ADL scores at admission	80 (60–95)	80 (60–95)	85 (60–100)	85 (60–100)	80 (55–95)	< 0.001
Blood pressure, mmHg						
SBP	149.61 ± 19.73	147.29 ± 18.71	149.13 ± 19.39	149.27 ± 18.63	152.75 ± 21.61	< 0.001
DBP	87.04 ± 12.66	86.81 ± 14.51	86.24 ± 11.51	86.76 ± 11.15	88.34 ± 13.03	< 0.001
**Medical history**						
Heart failure, *n* (%)	98 (2)	15 (1)	21 (2)	25 (2)	37 (3)	0.012
Previous heart disease, *n* (%)	1,095 (21)	232 (18)	267 (21)	266 (22)	330 (26)	<0.001
Diabetes mellitus, *n* (%)	1,616 (32)	488 (37)	401 (31)	363 (29)	364 (28)	<0.001
Hypertension, *n* (%)	3,458 (67)	883 (67)	847 (66)	803 (65)	925 (72)	0.002
Atrial Fibrillation, *n* (%)	340 (7)	43 (3)	75 (6)	86 (7)	136 (11)	<0.001
Hyperlipidemia, *n* (%)	779 (15)	169 (13)	190 (15)	188 (15)	232 (18)	0.004
Prior stroke, *n* (%)	1824 (36)	489 (37)	454 (35)	418 (34)	463 (36)	0.339
Toast type, *n* (%)						<0.001
LAA	3,995 (78)	1,046 (80)	1,002 (78)	953 (77)	994 (77)	
CE	304 (6)	42 (3)	60 (5)	79 (6)	123 (10)	
SAO	695 (14)	199 (15)	185 (14)	171 (14)	140 (11)	
SOE	135 (3)	25 (2)	42 (3)	32 (3)	36 (3)	

NIHSS, National Institute of Health Stroke Scale; mRS, modified Rankin scale; ADL, activities of daily living; LDH, lactate dehydrogenase; SBP, Systolic Blood Pressure; DBP, Diastolic Blood Pressure; LAA, large-artery atherosclerosis; CE: cardio-embolism; SAO: small-vessel occlusion; SOE: stroke of other determined etiology.

### Baseline characteristics by quartile of LDH

The median LDH concentration was 186 U/L (interquartile range 161–204.2). Detailed distribution of LDH was displayed in Supplementary Figure S2. Compared with patients in Q1 of LDH, those in Q4 of LDH were older (*p* < 0.001); and had a higher proportion of female and a lower proportion of male (*p* < 0.001); and had higher NIHSS scores at admission (*p* = 0.002) and lower ADL scores at admission (*p* < 0.001); and had higher systolic blood pressure and diastolic blood pressure (*p* < 0.001); and a higher prevalence of heart failure (*p* = 0.012), previous heart disease (*p* < 0.001), diabetes mellitus (*p* < 0.001), hypertension (*p* = 0.002), atrial fibrillation (*p* < 0.001), and hyperlipidemia (*p* < 0.001). A higher quartile of LDH was also positively associated with a higher prevalence of CE subtype of ischemic stroke (Table 1).

**Figure 3:**
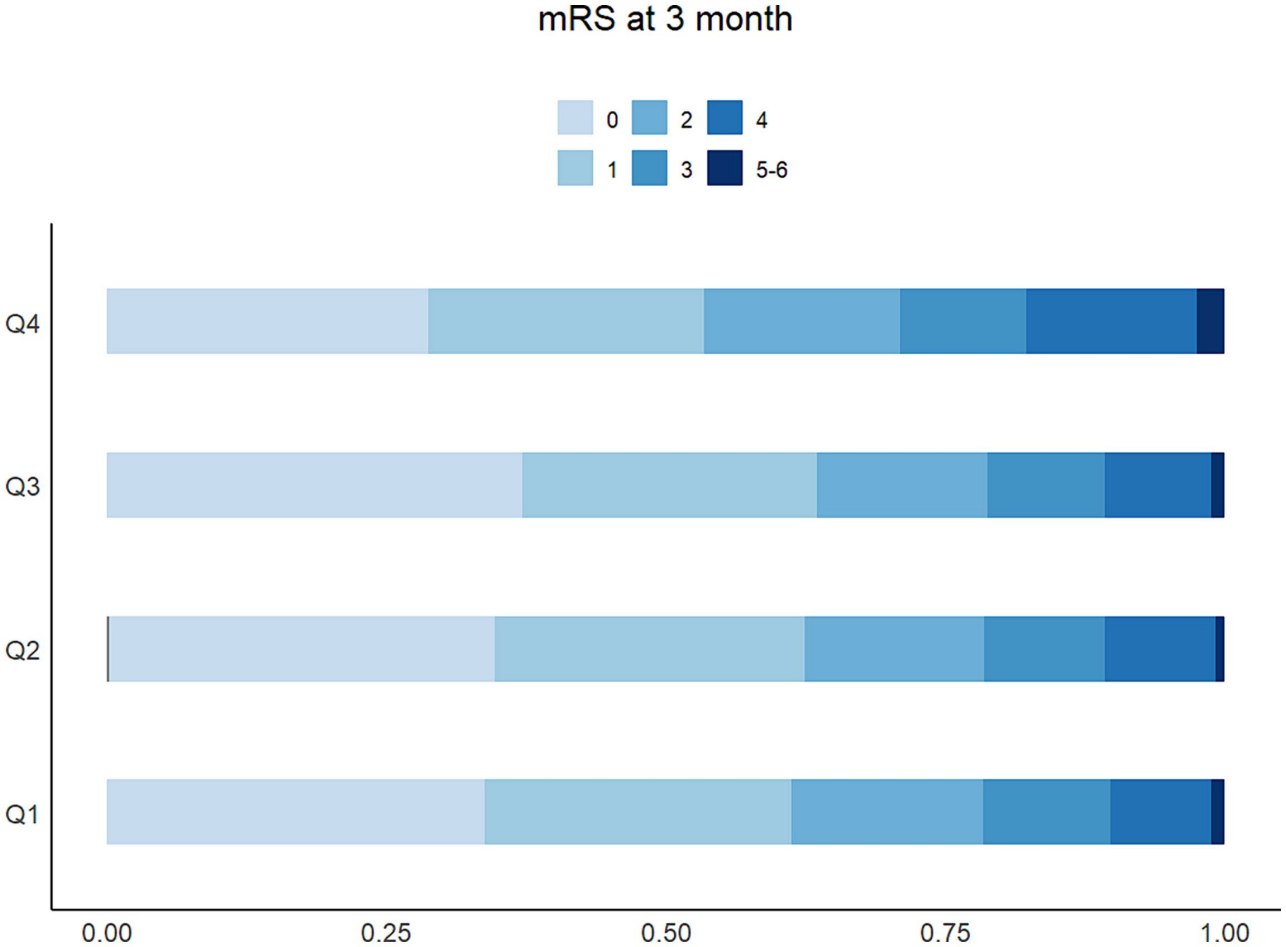
The distribution and proportion of mRS score at 90 days based on the degree of LDH among 5,129 patients.

**Figure 4:**
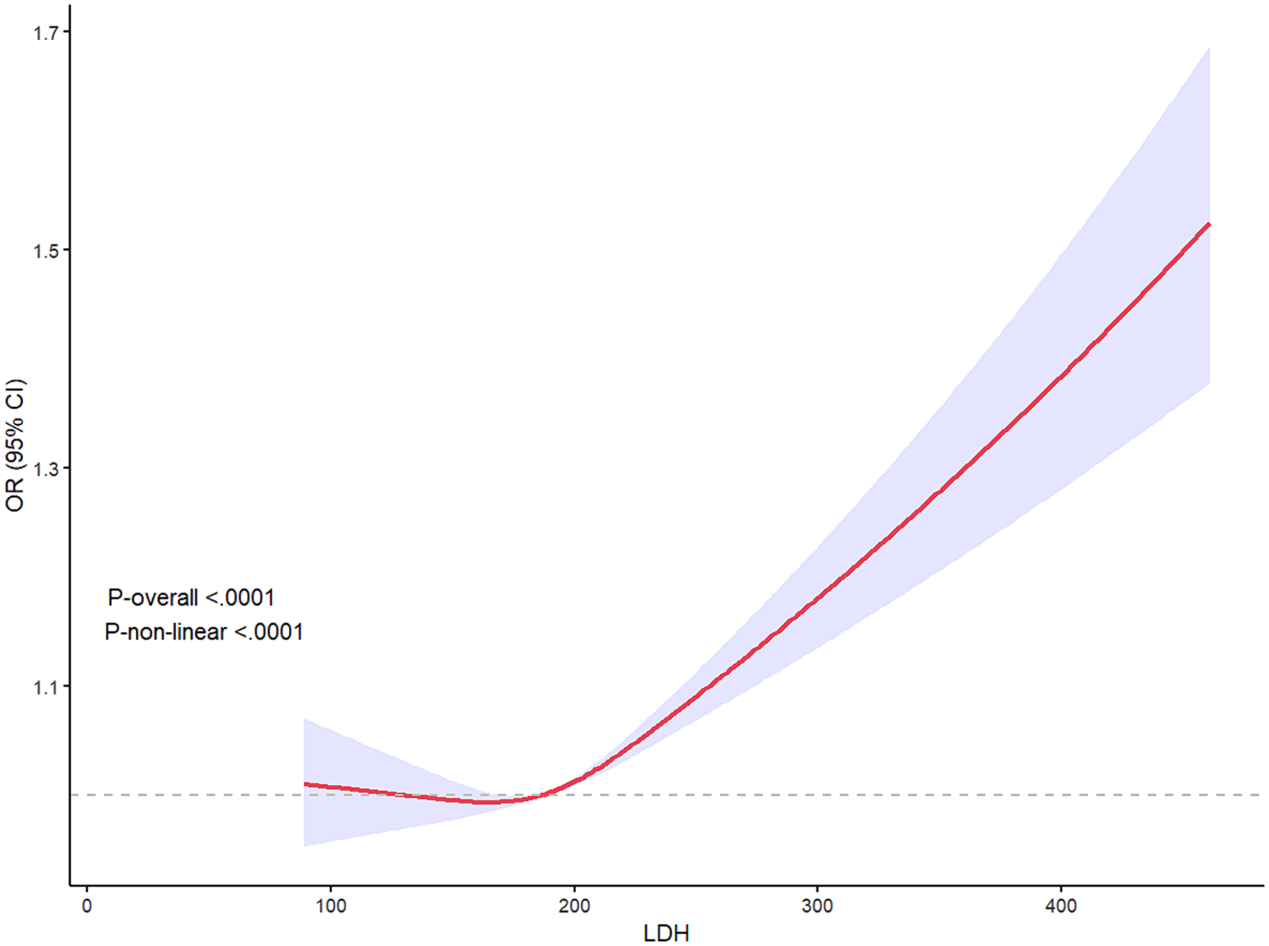
Restricted cube regression (RCS) presented the relationship between LDH and 90-day poor prognosis of AIS patients, illustrating the non-linear relationship between LDH and 90-day outcome (P-non-linear<0.001). LDH >200 seemed to be a risk factor for 90-day functional disability.

The proportion of patients with higher mRS scores (Gu et al., 2022; Shekhar et al., 2018; Jin X. X. et al., 2022; Wang et al., 2021) in Q4 of LDH was more than 25%, higher than any other groups, which was shown in the detailed distribution of 90-day mRS scores varying from 0 to 6 in Figure 3. The higher concentration of LDH (>200 U/L) was presented as a risk factor of 90-day functional disability of AIS patients, while the lower LDH concentration appeared to be stable (Figure 4). And the apparent critical point (200 U/L) in Figure 4 was roughly consistent with the fourth quartile (>204.2 U/L).

### Association between LDH, stroke recurrence and functional disability

A total of 1,200 (23.4%) and 371(7.2%) patients experienced functional disability and stroke recurrence during 90-day follow-up, respectively. Compared to patients with LDH levels in the lowest quartile, those with highest quartile of LDH levels tended to have a higher risk of functional disability (aOR 1.21, 95%CI, 1.00–1.47, *p* = 0.04) and a significantly higher risk of stroke recurrence (8.97% vs. 5.03%, aOR 1.21, 95%CI, 1.00–1.47, *p* < 0.001) at 90 days (Table 2). In addition, a 1-SD increase in LDH was associated with a 7% increase of the adjusted risk of functional disability (aOR 1.07, 95%CI 1.04—1.09, *p* < 0.001) and a 2% increase of the adjusted risk of stroke recurrence (aOR 1.02, 95%CI 1.01–1.04, *p* = 0.02), as shown in Table 2.

**Table 2:** Association between LDH and functional outcomes and stroke recurrence at 90 days.

Outcomes	No of patients	Events (%)	Unadjusted analysis		Adjusted analysis	
			Crude OR (95%CI)	Crude P	Adjusted OR (95% CI)	Adjusted P
Functional disability at 90 days (mRS > =2)						
Quartile 1	1,312	284 (21.6)	1.00(Reference)		1.00(Reference)	
Quartile 2	1,289	278 (21.6)	1.00 (0.83–1.20)	0.96	0.93 (0.76–1.13)	0.45
Quartile 3	1,235	262 (21.2)	0.97 (0.81–1.18)	0.76	0.88 (0.73–1.09)	0.25
Quartile 4	1,293	376 (29.1)	1.48 (1.24–1.77)	<0.001	1.21 (1.00–1.47)	0.04
Per SD			1.01 (1.003–1.011)	<0.001	1.07 (1.04–1.09)	<0.001
Stroke recurrence at 90 days						
Quartile 1	1,312	66 (5.03)	1.00(Reference)		1.00(Reference)	
Quartile 2	1,289	70 (5.43)	1.08 (0.59–1.28)	0.65	1.02 (0.71–1.44)	0.93
Quartile 3	1,235	81 (6.56)	1.31 (0.93–1.83)	0.12	1.22 (0.87–1.72)	0.26
Quartile 4	1,293	116 (8.97)	1.86 (1.36–2.54)	<0.001	1.21 (1.00–1.47)	<0.001
Per SD			1.00 (1.00–1.01)	<0.001	1.02 (1.01–1.04)	0.02

LDH indicates lactate dehydrogenase; OR, odds ratio. Adjusted for (functional outcome at 90 day) age, gender, smoking status, diabetes mellitus, hypertension, hyperlipidemia and toast type. Adjusted for (stroke recurrence) age, drinking, previous heart disease, hypertension, hyperlipidemia, toast type and Activities of Daily Living scores at admission.

### Correlation analysis of LDH and hs-CRP

The Pearson’s correlation analysis revealed a significant positive correlation between LDH and hs-CRP (*r* = 0.12, *p* < 0.001) among 1,259 patients that both tested LDH and hs-CRP. As shown in Figure 2, a total of 351 patients suffered functional disability, and the functional disability for those in the highest quartile (Q4) of LDH, in the highest quartile of hs-CRP (Q4), and the union of both Q4 groups were 107 (30.48%), 125 (35.61%) and 186 (52.99%), respectively (*p* < 0.05). In the cohort of 119 out of 351 individuals whose levels of LDH and hs-CRP were either in the Q1 or Q4, the functional disability for those within the LDH Q1 and hs-CRP Q1 group, the LDH Q1 and hs-CRP Q4 group, the LDH Q4 and hs-CRP Q1 group, and the LDH Q4 and hs-CRP Q4 group were 20 (16.81%), 36 (30.25%), 17 (14.28%) and 46 (38.65%), respectively (*p* < 0.05). Among those patients, functional disability rate in the union of both Q4 groups was up to 83.19% ((17 + 46 + 36)/119).

### Mediation effect of stroke recurrence

Among the 1,200 functionally disabled patients, 18% (*n* = 216) experienced stroke recurrence prior to the onset of functional disability. Table 3 delineates the total, direct, and indirect associations of LDH with functional disability. The indirect association, mediated through subsequent stroke recurrence, indicates an average 0.1% increase in the risk of functional disability development (adjusted odds ratio [aOR] 1.001; 95% CI, 1.00–1.005, *p* < 0.001). The proportion of the association between LDH levels and functional disability that is mediated by stroke recurrence is estimated to be 12.90% (95% CI, 6.22–21.16%, *p* < 0.001). After excluding patients who experienced mortality prior to stroke recurrence, the mediation effect through stroke recurrence was found to account for 11.48% (95% CI, 2.75–18.22%, *p* < 0.001) of the association between LDH levels and functional disability, as detailed in Table 3.

**Table 3:** Proportion of association of per SD of LDH with 90-day mRS mediated by follow-up stroke recurrence.

Effect	Unadjusted analysis		Adjusted analysis	
	Estimate (95%CI)	*P*	Estimate (95%CI)	*P*
Of 5,129 patients				
Total effect (TE), OR	1.01 (1.00–1.08)	<0.001	1.008 (1.00–1.009)	<0.001
Natural direct effect (NDE), OR	1.009 (1.00–1.01)	<0.001	1.005 (1.00–1.007)	<0.001
Natural indirect effect (NIE), or	1.001 (1.00–1.007)	<0.001	1.001 (1.00–1.005)	<0.001
Percentage mediated (PM)	13.11 (7.51–22.25)	<0.001	12.90 (6.22–21.16)	<0.001
Of 5,044 patients				
Total Effect (TE), OR	1.007 (1.00–1.009)	<0.001	1.006 (1.00–1.008)	<0.001
Natural direct effect (NDE), OR	1.006 (1.00–1.008)	<0.001	1.005 (1.00–1.008)	<0.001
Natural indirect effect (NIE), OR	1.001 (1.00–1.004)	<0.001	1.001 (1.00–1.005)	<0.001
Percentage mediated (PM)	11.27 (5.7–18.13)	<0.001	11.48 (2.72–18.22)	<0.001

LDH indicates lactate dehydrogenase; OR, odds ratio. Adjusted for age, gender, smoking status, diabetes mellitus, hypertension, hyperlipidemia and toast type.

Table 4 presents a detailed analysis of the total, direct, and indirect associations between LDH or hs-CRP and functional disability among 1,258 patients. Within this patient group, a subset of 351 individuals experienced functional disability, with 7.4% (*n* = 26) suffering a stroke recurrence as a precursor to their functional disability. Specifically, the mediated effects through stroke recurrence on the association between levels of LDH and hs-CRP with functional disability are calculated at 10.23% (95% CI, 1.85 to 87%, *p* < 0.001) and 13.81% (95% CI, 3.22 to 31.28%, *p* < 0.001), respectively.

**Table 4:** Proportion of association of per SD of LDH or hs-CRP with 90-day mRS mediated by follow-up stroke recurrence.

Effect	Unadjusted analysis		Adjusted analysis	
	Estimate (95%CI)	*P*	Estimate (95%CI)	*P*
Of 1,258 patients				
LDH				
Total effect (TE),OR	1.0007 (1.00–1.0008)	<0.001	1.0005 (1.00–1.001)	<0.001
Direct effect (NDE),OR	1.0006 (1.00–1.0007)	<0.001	1.0004 (1.00–1.001)	<0.001
Indirect effect (NIE),OR	1.0001 (1.00–1.0005)	<0.001	1.00006 (1.00–1.0008)	<0.001
Percentage Mediated (PM)	12.01 (3.06–28.12)	<0.001	10.23 (1.85–87.00)	<0.001
HS-CRP				
Total effect (TE),OR	1.003 (1.001–1.008)	<0.001	1.001 (1.00–1.002)	<0.001
Direct effect (NDE),OR	1.002 (1.001–1.007)	<0.001	1.002 (1.00–1.008)	<0.001
Indirect effect (NIE),OR	1.0003 (1.00–1.006)	<0.001	1.0003 (1.00–1.007)	<0.001
Percentage Mediated (PM)	12.61 (3.25–27.14)	<0.001	13.81 (3.22–31.28)	<0.001

LDH indicates lactate dehydrogenase; hs-CRP indicates hyper sensitive C-reactive protein; OR, odds ratio. Adjusted for age, gender, smoking status, diabetes mellitus, hypertension, hyperlipidemia and toast type.

### Sensitivity analysis of mediation analysis

We also calculated the estimates of direct and indirect associations among patients excluding death patients before stroke recurrence to avoid the influence of competing risk of death. Results showed that stroke recurrence mediated 11.48% (95%CI 2.72–18.22%, *p* < 0.001) of the association between LDH and functional disability. Data across major strata defined by age (≤65,>65), smoking (yes, no), gender (female, male), smoking status (yes, no), heart failure (yes or no), drinking (yes, no), previous heart disease (yes, no), hypertension (yes, no), atrial fibrillation (yes, no), ischemic heart disease (yes, no), hyperlipidemia (yes, no), prior stroke (yes, no), TOAST type (LAA, CE, SAO, SOE), mRS scores at admission (<3, ≥3), NIHSS scores at admission (<3, ≥3) and ADL scores at admission (≤60, >60) showed comparable results (Figure 4).

## Discussion

**Figure 5:**
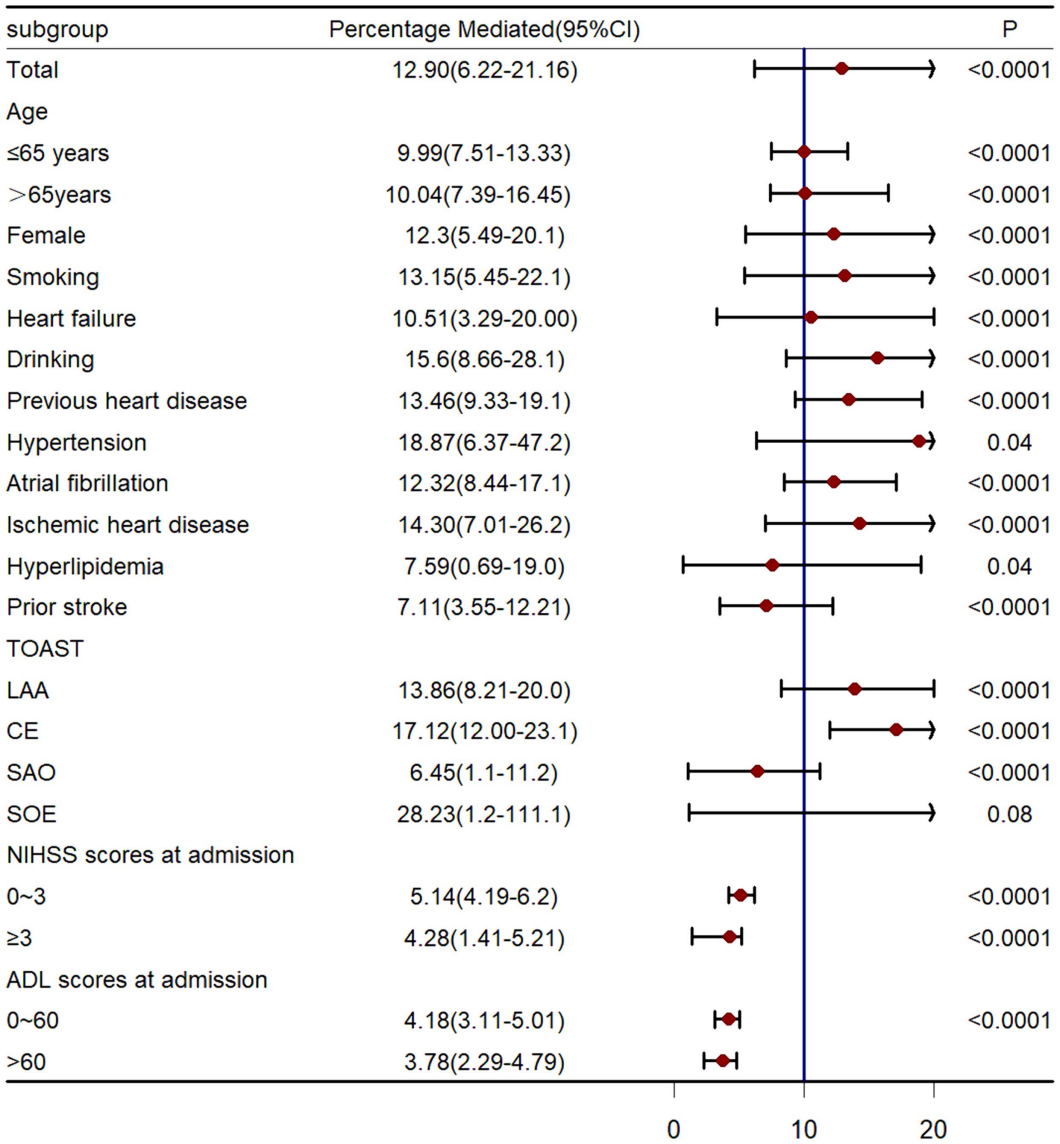
Causal mediation analysis stratified by prespecified subgroup. LAA, large-artery atherosclerosis; CE, cardio-embolism; SAO, small-vessel occlusion; SOE: stroke of other determined etiology.

In the current cohort study, our mediation analysis indicated that the relationships between LDH or hs-CRP levels and functional outcomes at 90 days were only partially mediated by stroke recurrence. Specifically, less than 20% of the variability in functional outcomes, as measured by mRS scores, could be attributed to differences in stroke recurrence. Both LDH and hs-CRP are crucial predictors of functional outcomes and recurrence in AIS patients. These findings indicate that traditional secondary prevention strategies targeting stroke recurrence may be insufficient for improving functional independence within this population. Further investigation into novel anti-inflammatory therapies is warranted and more attention to LDH should be given in AIS patients (see Figure 5).

LDH, as an intracellular inflammation factor, is known to increase in patients with central nervous system disorders such as AIS (Lampl et al., 1990) as a result of brain cell damage or death. Several researches have demonstrated a correlation between LDH levels and functional disability or stroke recurrence following ischemic stroke. For instance, evidence from a subgroup analysis of the CNSR-III (Third China National Stroke Registry) trial suggested that elevated LDH levels are linked to unfavorable outcomes in patients with ischemic stroke (Wang et al., 2021). Data from Jin et al. showed that elevated LDH was linked to stroke recurrence of AIS patients (Jin et al., 2022). Additionally, given the limited research on LDH as a predictor of functional disability in AIS patients, we conducted a preliminary analysis to assess LDH’s predictive value for 90-day functional outcomes. Our ROC curve analysis yielded comparable results, suggesting that LDH may serve as a risk factor in this context (Supplementary Figure S3). Consistent with prior literature, current study found a positive correlation between LDH levels and the likelihood of stroke recurrence and functional disability.

At present, numerous studies in the literature examine the role of hs-CRP as a prognostic marker in patients with AIS, with findings suggesting that hs-CRP may serve as a potential predictor of functional outcomes and recurrence risks (Cheng et al., 2023; Pu et al., 2022; Li et al., 2016). Conversely, research focusing on serum LDH as a prognostic marker in AIS, either in isolation or in conjunction with elevated hs-CRP levels, remains relatively limited (Sharma et al., 2021). In present study, we observed a positive correlation between LDH levels and hs-CRP levels; the functional outcome rate was exceeded 50% among individuals in the combined fourth quartiles (Q4) for both measures. The effect mediated by stroke recurrence was similar for both markers, each accounting for less than 20%, suggesting that the role of LDH in predicting recurrence and functional outcome in AIS patients should not be underestimated and assessing and improving the prognosis of AIS patients may reap greater benefits by considering the combined test results of LDH and hs-CRP.

Evidence suggested that AIS events may lead to neuronal damage and functional disability (Xu et al., 2022; Tuo et al., 2022; Wardlaw et al., 1998; Rohweder et al., 2015; Meyer et al., 2015). Our study found a correlation between LDH levels and both functional disability and stroke recurrence. We proposed that stroke recurrence acts as a mediator in the relationship between LDH levels and functional disability, with the mediation effect estimated through mediation analysis. To the best of our knowledge, no extant research has investigated the potential mediating effect of stroke recurrence on the relationship between LDH levels and functional disability. This study seeks to elucidate the underlying mechanisms contributing to functional disability post-stroke, with a specific focus on the potential advantages of integrating anti-inflammatory therapy with conventional anti-thrombotic, anti-hypertensive, and lipid-lowering treatments to improve functional outcomes following a stroke.

Some previous studies have indicated that the impact of proinflammatory biomarkers such as hs-CRP (Gu et al., 2022) and interleukin-631 on functional disability following stroke is primarily mediated by a less than 20% contribution from recurrent strokes, which were strengthened by our results. The intricate relationship between inflammation and functional outcomes post-stroke is not yet fully understood, as it involves a complex interplay of interconnected molecular pathways and cellular mechanisms (Pei et al., 2015; Gu et al., 2022; Pawluk et al., 2020). Several studies have demonstrated that elevated levels of LDH can lead to increased lactic acid production (Dick et al., 2016), inhibiting cytotoxic T lymphocytes which contributes to the brain recovery (Wang S. et al., 2016; Wang et al., 2017), and enhance the inflammatory response induced by lipopolysaccharides within macrophages (Dick et al., 2016). Evidences clearly support that inflammation is a contributing factor to cell death, brain injury, and disruption of the blood–brain barrier, ultimately leading to an increase in cerebral infarct size and further functional impairment (Doyle et al., 2008; Corps et al., 2015).Moreover, inflammation is a significant factor in the development and advancement of atherosclerosis, plaque rupture, platelet aggregation, and intravascular thrombosis, all of which elevate the likelihood of stroke and subsequent functional impairment (Milias et al., 2005; Fattizzo et al., 2022).

In contemporary secondary clinical interventions for stroke, traditional therapies such as antiplatelet (Yedavalli et al., 2024), antihypertensive, lipid-lowering, and hypoglycemic agents are commonly utilized, while anti-inflammatory strategies are frequently overlooked (Gu et al., 2023). Our analysis indicates that stroke recurrence accounts for less than 20% of the variance in functional outcomes, suggesting that over 80% of functional damage is attributed to the pathway between LDH and disability independent of stroke recurrence. Consequently, anti-inflammatory therapy warrants more attention (Gu et al., 2022, 2023). LDH has been identified as a potential biomarker for inflammatory burdens, and its inhibitors may be utilized for anti-inflammatory purposes (Manerba et al., 2017; Miyoshi et al., 2018). Given the limited research on LDH as a biomarker for stroke, it is imperative to conduct experimental and clinical investigations in carefully selected high-risk subjects.

Several limitations were present in this study. Firstly, it should be noted that this study was conducted retrospectively using data from a single center’s prospective database, potentially limiting its ability to accurately represent the broader population. The ROC curve analysis of LDH in predicting 90-day functional outcomes was also conducted using data from the single database. Our findings suggest that LDH may serve as a potential prognostic indicator for poor outcomes in AIS patients, offering a relatively novel perspective for further research. Additional studies on LDH in the context of ischemic stroke are encouraged to confirm and expand upon these results. Second, certain eligible participants were excluded from the study due to missing data on LDH or follow-up results, potentially leading to selection bias. However, a comparison of baseline characteristics between included and excluded participants revealed mostly comparable results (Supplementary Table S2). And the primary outcomes of utmost importance, specifically LDH concentration, 90-days mRS scores and stroke recurrence of all patients, were fully documented, ensuring the accuracy and credibility of the study. Third, the baseline characteristic of prior stroke in fourth subgroups (Table 1) exhibited no significant association with balance (*p* > 0.05), which may introduce confounding bias. However, results of mediation analysis stratified by prior stroke showed comparable mediation effects, which indicated that mediation effects would not be affected by prior stroke and the analysis of prior stroke as a mediating variable helped to reduce confounding bias and facilitate a more precise evaluation of the impact of LDH concentration on the outcomes. Forth, based on the comprehensive analysis of LDH distribution as depicted in Supplementary Figure S2, it was observed that LDH exhibited a skewed distribution, potentially introducing bias in the estimation of mediating effects. Nevertheless, sensitivity analysis conducted on the per-SD of the log-transformed LDH values yielded consistent and reliable results. Fifth, our study did not address the specific correlation between peripheral blood LDH levels and the degree and time of brain cell damage, a shortfall attributable to the absence of pertinent data. Collection of data regarding the interval from stroke onset to hospital admission for LDH assessment, alongside the determination of stroke severity, would enable the execution of more refined research investigating the association between LDH levels and the recurrence of stroke as well as the occurrence of poor prognostic outcomes in stroke patients. Sixth, despite meticulous control of potential covariates in assessing the mediating effect, the presence of unmeasured confounders cannot be completely ruled out. Ultimately, while the mRS is widely recognized and validated, it is important to acknowledge that it is a subjective measure derived from patient reports. Further research may be necessary to fully understand the extent to which mRS scores accurately capture functional changes.

## Conclusion

In conclusion, our findings indicate that stroke recurrence accounts for less than 20% of the mediating effect on the association between LDH levels and functional outcomes at 90 days in patients with AIS, suggesting that there is substantial room for novel anti-inflammatory interventions, particularly those targeting LDH inhibition, which may significantly enhance the functional recovery of AIS patients. Equal consideration should be given to the roles of LDH and hs-CRP in the prognosis and recurrence of AIS patients, as a combined assessment of both biomarkers is likely to yield greater benefits.

## Data Availability

The raw data supporting the conclusions of this article will be made available by the authors, without undue reservation.
