# Supplementary material for: Mediation effect of stroke recurrence in the association between post-stroke lactate dehydrogenase and functional disability
Source: Front Aging Neurosci. 2024 Aug 30;16:1450863. doi: 10.3389/fnagi.2024.1450863 (PMC11392875; doi:10.3389/fnagi.2024.1450863)
Supplement: Supplementary file 1 [file Data_Sheet_1.PDF]

Supplementary Materials:

Table S1: The baseline characteristics comparison between original data and imputed data.

Variables	Original data (n = 1235)	Imputed data (n = 1293)	*P* value
Demographic			
Age	64 (58, 71)	64 (58, 71)	1.00
Gender, n (%)			1.00
Male	3632 (71)	3632 (71)	
Female	1497 (29)	1497 (29)	
Smoking, n (%)	2232 (44)	2234 (44)	0.99
Drinking, n(%)	1503 (29)	1503 (29)	1.00
NIHSS scores at admission	3.0(2.0-5.0)	3.0(2.0-5.0)	1.00
ADL scores at admission	80(60-95)	80(60-95)	1.00
Blood pressure, mmHg			
SBP	148 (138, 160)	148 (138, 160)	1.00
DBP	85 (80, 94)	85 (80, 94)	1.00
Medical history			
Heart failure, n (%)	95 (2)	98 (2)	0.935
Previous heart disease, n (%)	1095 (21)	1095 (21)	1.00
Diabetes mellitus, n (%)	1616 (32)	1616 (32)	1.00
Hypertension, n (%)	3458 (67)	3458 (67)	1.00
Atrial Fibrillation, n (%)	340 (7)	340 (7)	1.00
Hyperlipidemia, n (%)	720 (14)	720 (14)	1.00
Prior stroke, n (%)	1823 (36)	1824 (36)	1.00
Toast type, n (%)			1.00
LAA	3995 (78)	3995 (78)	
CE	304 (6)	304 (6)	
SAO	695 (14)	695 (14)	
SOE	135 (3)	135 (3)	

Abbreviations: NIHSS, National Institute of Health Stroke Scale; mRS, modified Rankin scale; ADL, activities of daily living; LDH, lactate dehydrogenase; LAA, large-artery atherosclerosis; CE: cardio-embolism; SAO: small-vessel occlusion; SOE: stroke of other determined etiology.

Table S2: The baseline characteristics comparison between excluded patients and included patients.

Variables	Excluded patients (n = 2909)	Included patients (n = 5129)	*P* value
Demographic			
Age	66 (59, 73)	65 (58, 72)	<0.001
Gender, n (%)			0.125
Male	2017 (69)	3641 (71)	
Female	892 (31)	1488 (29)	
Smoking, n (%)	1415 (49)	2242 (44)	<0.001
Drinking, n(%)	849 (29)	1499 (29)	0.99
NIHSS scores at admission	3.0(1.0-6.0)	3.0(2.0-5.0)	0.25
ADL scores at admission	80(60-95)	80(60-100)	0.06
Medical history			
Heart failure, n (%)	86 (3)	101 (2)	0.006

Previous heart disease, n (%)	674 (23)	1089 (21)	0.047
Diabetes mellitus, n (%)	918 (32)	1620 (32)	0.99
Hypertension, n (%)	1958 (67)	3436 (67)	0.79
Atrial Fibrillation, n (%)	212 (7)	334 (7)	0.2
Hyperlipidemia, n (%)	402 (14)	731 (14)	0.615
Prior stroke, n (%)	1045 (36)	1816 (35)	0.66
Toast type, n (%)			<0.001
LAA	2249 (77)	3992 (78)	
CE	104 (4)	309 (6)	
SAO	463 (16)	693 (14)	
SOE	93 (3)	135 (3)	

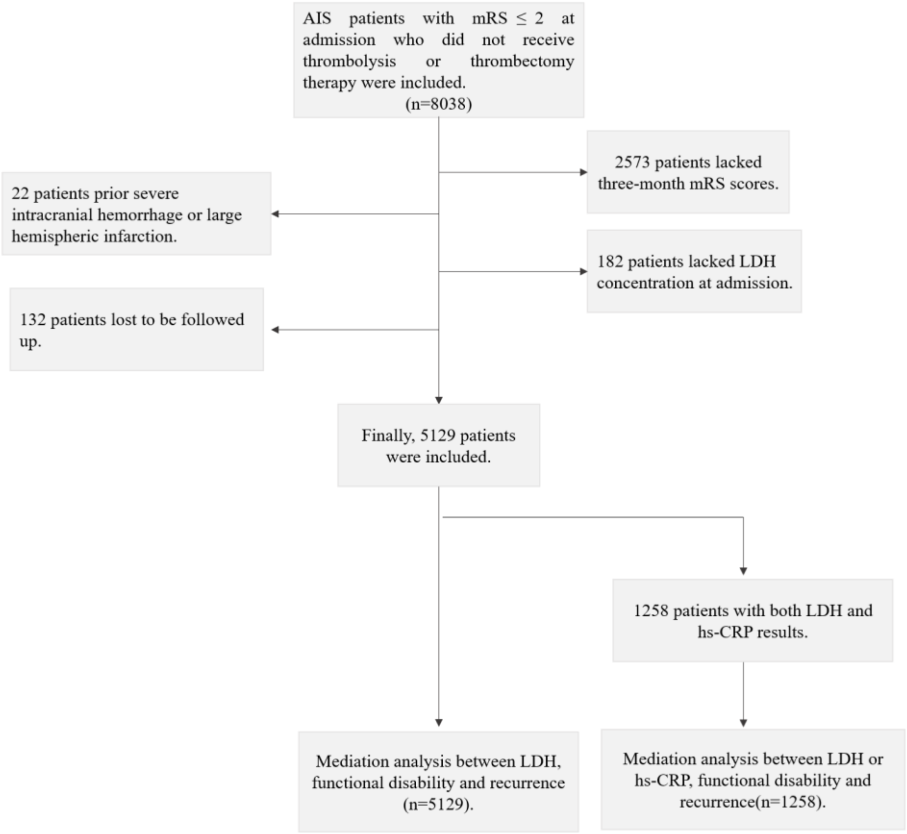

Figure S1: Flowchart for this analysis.

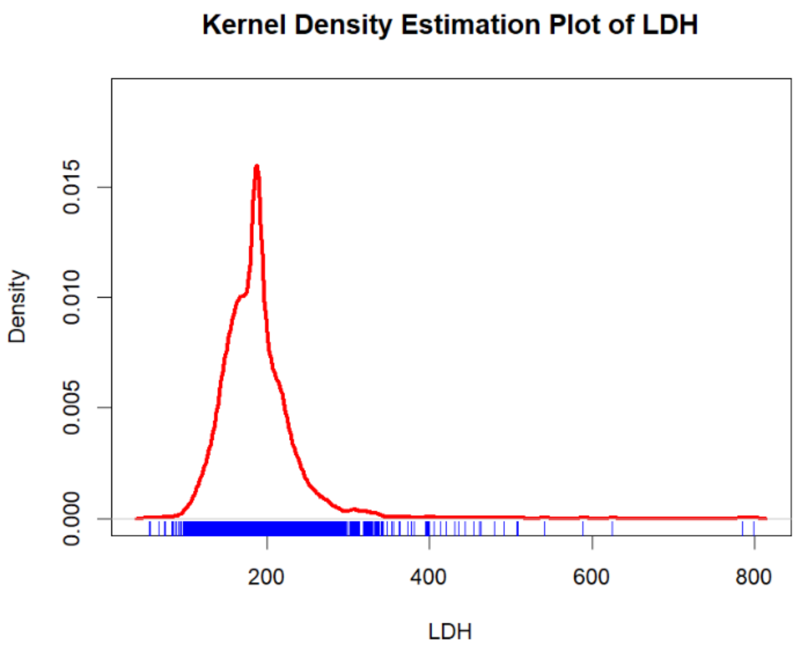

Figure S2: Detailed distribution of LDH in kernel density estimation plot.

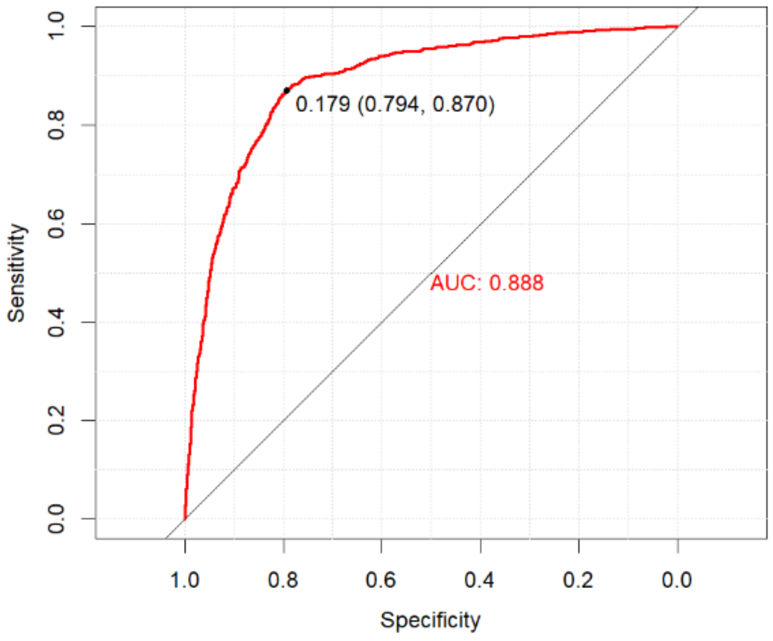

Figure S3: Receiver operating characteristic (ROC) curves of LDH with several covariates (age, gender, smoking status, diabetes mellitus, hypertension, hyperlipidemia and toast type) predicting three-month adverse outcome. The nomogram had good discriminative power with an area under the ROC curve(AUC) of 0.888 (95% CI, 0.830-0.905).
